# Chaos in Balance: Non-Linear Measures of Postural Control Predict Individual Variations in Visual Illusions of Motion

**DOI:** 10.1371/journal.pone.0113897

**Published:** 2014-12-02

**Authors:** Deborah Apthorp, Fintan Nagle, Stephen Palmisano

**Affiliations:** 1 Research School of Psychology, College of Medicine, Biology & Environment, Australian National University, Canberra, Australian Capital Territory, Australia; 2 School of Psychology, Faculty of Social Sciences, University of Wollongong, Wollongong, New South Wales, Australia; 3 Centre for Mathematics, Physics and Engineering in the Life Sciences and Experimental Biology, University College London, London, United Kingdom; UMR8194, France

## Abstract

Visually-induced illusions of self-motion (vection) can be compelling for some people, but they are subject to large individual variations in strength. Do these variations depend, at least in part, on the extent to which people rely on vision to maintain their postural stability? We investigated by comparing physical posture measures to subjective vection ratings. Using a Bertec balance plate in a brightly-lit room, we measured 13 participants' excursions of the centre of foot pressure (CoP) over a 60-second period with eyes open and with eyes closed during quiet stance. Subsequently, we collected vection strength ratings for large optic flow displays while seated, using both verbal ratings and online throttle measures. We also collected measures of postural sway (changes in anterior-posterior CoP) in response to the same visual motion stimuli while standing on the plate. The magnitude of standing sway in response to expanding optic flow (in comparison to blank fixation periods) was predictive of both verbal and throttle measures for seated vection. In addition, the ratio between eyes-open and eyes-closed CoP excursions during quiet stance (using the area of postural sway) significantly predicted seated vection for both measures. Interestingly, these relationships were weaker for contracting optic flow displays, though these produced both stronger vection and more sway. Next we used a non-linear analysis (recurrence quantification analysis, RQA) of the fluctuations in anterior-posterior position during quiet stance (both with eyes closed and eyes open); this was a much stronger predictor of seated vection for both expanding and contracting stimuli. Given the complex multisensory integration involved in postural control, our study adds to the growing evidence that non-linear measures drawn from complexity theory may provide a more informative measure of postural sway than the conventional linear measures.

## Introduction

The sensation of self-motion induced by large-field visual stimuli (known as ‘vection’; [1], [2]) can be quite compelling, yet there are large individual variations in the experience of this phenomenon. Since these variations might have significant real-world implications (e.g. susceptibility to motion sickness, accuracy in virtual driving/aviation environments, etc.), it would be useful to have some insight into the underlying causes. One possible predictor is visual control of posture: that is, the extent to which people rely on visual cues to maintain steady upright posture. While much study has examined postural control in the areas of ageing [3], [4], balance-related disorders such as Parkinson's Disease [5], [6], and, in some cases, multisensory integration [7]–[9], few studies have examined its role in self-motion perception.

### Effects of optic flow on posture

Several groups have examined the effect of visual scene motion on postural readjustment [10]–[12]; the relationship is not straightforward, and most models assume some kind of continuous, non-linear multisensory feedback system (e.g. see [13], [14]). A recent paper examined the role of perceptual uncertainty in visual flow fields [15], concluding that near-optimal sensory weighting under a simple Bayesian model [16], [17] was sufficient to explain the results. However, although somewhat misleadingly including the word “vection” in the title, the authors did not actually measure vection itself.

The relationship between visually-induced postural sway and vection has been less well examined; Tanahashi et. al. [18] suggested that both phenomena might be underpinned by the same basic mechanisms. They found that subjects exhibited greater postural disturbances when vection was experienced during visually simulated roll motion (indicated by a button-press), compared to no vection. Postural disturbances were still evident to the stimuli when vection was not experienced, but these were smaller, and the authors suggest that the two phenomena might merely have different thresholds.

Guerraz and Bronstein [19] explored this notion further by utilising stimuli that could evoke postural responses in either the same or opposite direction to the simulated visual motion, and exploring both the vection and postural responses to these stimuli. In this study, a horizontally translating background checkerboard pattern was presented behind either a ground-fixed or head-fixed frame. With the ground-fixed frame, postural responses were (transiently) in the opposite direction to the background motion, while with the head-fixed frame, postural responses were only in the same direction as background motion. However, vection was only ever in one direction (opposite to the background motion, in the same direction as the simulated self-motion, as it almost always is). The ground-fixed frame provided motion parallax, which should lead to better vection, while the head-fixed frame provided no motion perspective, and so should lead to weaker vection. Consistent with this, in the head-fixed (compared to the ground-fixed) condition, vection developed considerably later, was reported less consistently, and was of shorter duration. The authors postulate two mechanisms, a shorter-latency system responsible for automatic postural adjustments, and a longer-latency system involved in postural adjustment in response to the conscious perception of self-motion.

However, neither of these studies examined vection magnitude; the relationship between vection magnitude and sway magnitude might shed some light on the relationship between these mechanisms.

### Effects of optic flow on motion sickness

Smart and Stoffregen [20] showed that visually induced motion sickness (when the environment, a moving room, oscillates at frequencies around 0.017 to 0.4 Hz, thought to interfere with the waveforms of normal postural sway) was preceded by, and predicted by, the variance in individual postural sway. In short, individuals who showed greater postural sway while exposed to the swaying room were more likely to experience motion sickness. They were also more likely to have more frequent and longer sessions of vection, but the paper does not dwell on this relationship. Another underplayed aspect of this study is that people who showed greater instability when sway was measured with eyes closed (thus in the absence of visual stimulation) were more likely to become sick. Were they also more likely to experience vection? Although the data would speak to this, the relationship is not explored at all. The authors cite another paper [21] where the relationship between vection and postural sway was explored. But this paper only explores the correlation between self-reported vection and magnitude of postural sway; it does not explore whether those who show greater postural variation in the first place are more prone to experiencing vection. This is a useful question which is yet to be fully explored.

### Postural control and vection

Palmisano et al. [12] measured the effect of jittering and non-jittering radially expanding and contracting optic flow on postural sway and on vection (in separate experiments for sway and vection). The horizontal and vertical simulated viewpoint jitter added to these radial flow displays was similar to camera shake. These results showed that jitter (in comparison to smooth motion) increased backwards sway in response to expanding flow, but **decreased** forwards sway in response to contracting flow; however, jitter increased **vection** in both directions. The authors measured both anterior-posterior (AP) and medial-lateral (ML) sway, but only AP sway showed the effects. A postural sway aftereffect was also seen in both directions. Variability was not reported, nor was sway with eyes closed.

More recently, Palmisano et al. [22] found that a measure of spontaneous standing sway (specifically, the Romberg Ratio, which measures path length of eyes-closed standing sway divided by eyes-open path) significantly predicted the vection experienced subsequently when standing in front of a large-field vection-inducing display. However, this measure only predicted vection for smooth radial flow displays, not for (vertically) oscillating radial flow displays (which produced stronger vection). Continuous monitoring of vection strength while standing can be problematic; any measure which requires manual activity, such as a button-press, can disrupt postural responses and thus also activity on the retina; thus, only verbal measures were used in this study, collected after each optic flow period. It is still a question of interest whether measures of visual control of posture can predict seated vection, where the vestibular input (given by the necessity to remain upright while viewing vection-inducing stimuli), as well as other proprioceptive inputs, would be less salient, and thus visual influences on self-motion perception might be greater.

### Measures of postural fluctuations

Postural fluctuations can be measured using a variety of different techniques, some more straightforward than others. Below we briefly outline some of the more common measures, and their advantages and disadvantages.

#### Linear measures

We use the term “linear measures” here to refer to those which assume the output of the system is directly proportional to the input. The simplest of these measures is path length. This involves computing the distance covered by the CoP over a certain time period by summing the Euclidean distances between points:
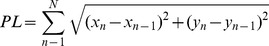
(1)where 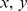 are the coordinates of the CoP and *N* is the number of data points [23].

This is one of the more common measures used, perhaps owing to its simplicity, and we have used a variant of this measure (the ratio between eyes-open and eyes-closed paths, as described above) to successfully predict standing vection [22].

Sway area is also commonly used, usually computed as a 95% confidence ellipse around the area covered by the CoP, computed using the eigenvalues of the variance/covariance matrix ([24], [25]; see Methods for details).

Other linear measures include those such as standard deviation and sway magnitude (anterior-posterior range). For a more complete review of some of these techniques, see [26].

#### Non-linear measures

Recently, a growing body of literature has addressed the notion that, since postural stability is achieved via the interaction of a number of different systems, both within and between senses (for instance, visual and vestibular - [27], [28]), the resulting measurements may be inherently nonlinear, and thus might be best investigated via analyses based on nonlinear dynamical approaches [29], [30]. Approaches to this include (but are not limited to) recurrence quantification analysis [3], [31], [32], wavelets [33] and detrended fluctuation analysis [34], [35].

Arguably the simplest of these methods is recurrence quantification analysis (RQA). This method was developed to quantify the number and duration of recurrences in a dynamical system, based on its phase space trajectory [25], [36]; it has been used in applications as diverse as economics, astrophysics, engineering, geophysics, physiology (in particular for heart rate variability), as well as in neuroscience (in particular for EEG data) [37]–[42]. Recently it has emerged as one of the more effective methods of quantifying recurrent fluctuations in postural sway during quiet standing [3], [31], [43]. Centre of pressure data tends to be non-stationary (meaning that local measures, such as joint distribution or mean, vary over time [3]; essentially, stationary data is locally self-similar over time), and RQA is applicable to non-stationary data [44].

**Figure 1:**
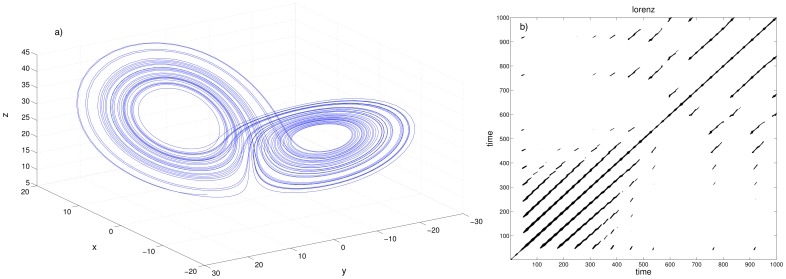
An illustration of the concept of recurrence plots, using the Lorenz system (a well-known 3-dimensional non-linear system - reproduced here from [68]). a) The Lorenz attractor: an example trajectory of the Lorenz system represented in 3-dimensional phase space. b) The recurrence plot for this trajectory. Both axes represent time. Looking along the *x* axis, we can follow the system's evolution. If the system's position in phase space at 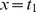 is closely approached at 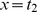, we place a dot at coordinates 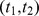. The positions at 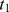 and 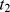 need not be exactly the same, but they must be close to within a tolerance 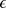 which we set to be very small. The recurrence plot thus shows all time points when the system returns very close to a previous state; each dot in the graph represents a revisit, and we can read the two visiting times from the *x* and *y* axes. Note that recurrence plots are symmetric. Code for reproducing these figures can be found at http://people.physik.hu-berlin.de/schinkel/timely/html/index.html.

RQA aims to uncover meaningful structure in postural fluctuations by exploring the recurrent patterns in the time series data produced by quiet standing CoP data. It involves producing recurrence plots showing pairs of times at which a system revisits previous positions, as well as numerous scalar measures, such as recurrence rate. For a detailed explanation of the underlying theory, see [45] and [46]. A graphical illustration of the concept is provided in Figure 1.

The basic principle behind RQA is that the phase space of a single time series can be reconstructed using time delay embedding. We build a vector 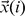 consisting of point 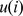 and *m* subsequent points spaced by 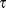: 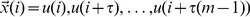
(2)


Here 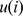 represents the time series (such as, in this case, movement along the anterior-posterior axis over time), *m* represents the embedding dimension and 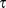 the time delay. It should be pointed out that the analysis is quite sensitive to each of these parameters, and care must be taken in selecting them [46], [47].

RQA produces a number of measures of the complexity of a system, the simplest of which is recurrence rate:
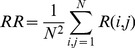
(3)


This represents the probability that any state will recur, and is represented by the density of points in the recurrence plot. Also available from the analysis are percent determinism (DET), laminarity (LAM), average line length (L), trapping time (*TT*), Shannon entropy (ENTR) and trend. However, for the purposes of simplicity in this paper we intend to focus on recurrence rate.

## Results

### Sway path during quiet stance and seated vection

**Figure 2:**
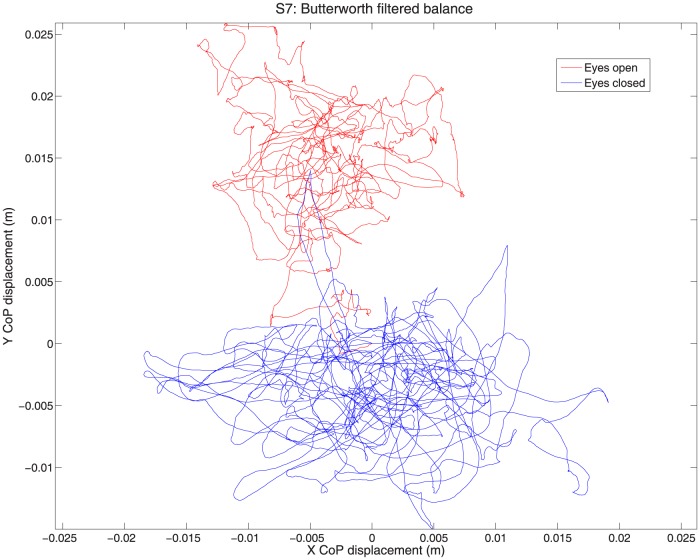
Quiet stance sway path for a single representative subject. The figure shows sway with eyes open (red) and eyes closed (blue) over a 60 second period. It should be pointed out that, according to traditional conventions, negative y values represent forwards postural sway.

For this analysis, we examine two of the main measures collected: postural sway during quiet stance, and subjective vection ratings (throttle or verbal) when seated. During quiet stance, almost all subjects exhibited greater postural sway with eyes closed than with eyes open. Initially, total sway path was calculated as the total distance travelled by the CoP over a 60-second period, using Equation 1. See Figure 2 for example data for a representative subject. The sway path was longer with eyes closed (mean  = 1.23 m) than with eyes open (mean  = .68 m); this difference was significant, t(12)  = 7.651, p<.001.

During seated vection, subjects gave quite variable vection ratings to contracting and expanding stimuli. Ratings were collected both via verbal reports (a percentage rating), and continuous monitoring with a throttle device (see Methods for details). The throttle data yielded both maximum measure (throttle max), and latency (number of seconds until onset of vection - measured by time until the online throttle rating reached a cutoff of 5 %). Means and standard deviations of these ratings are shown in Table 1. Verbal vection measures were significantly higher for contracting than expanding flow, *t*(12)  = 3.54, p = 0.004, but this difference was not significant for throttle maximum (p = 0.069) or latency measures (p = .502). We computed the Romberg Ratio for sway path (closed path/open path) for each individual. However, the Romberg Ratio did not show a significant relationship with any of the seated vection measures, unlike the relationship shown with standing vection in our previous study [22].

**Table 1:** Means and standard deviations for seated vection.

Measure	Expanding	Contracting
Verbal (%)	37.6 (21)	60.62 (27)
Throttle max (%)	33.24 (22)	42.44 (31)
Latency (s)	4.08 (2.4)	3.78 (1.9)

Means and standard deviations (in brackets) for the verbal, throttle maximum, and latency measures for expanding and contracting seated vection.

### Sway area ratios during quiet stance and seated vection

**Figure 3:**
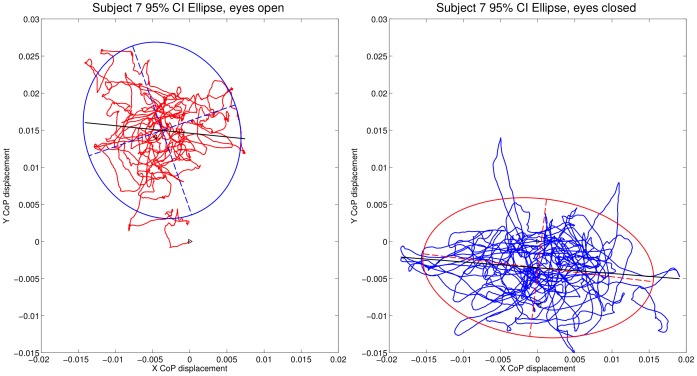
Ellipse fits for eyes open compared to eyes closed conditions for another representative subject. The figure shows sway with eyes open (red) and eyes closed (blue) over a 60 second period. The area ratio was calculated as the ratio of eyes-open to eyes-closed ellipse areas. Code for these calculations can be found at http://dx.doi.org/10.6084/m9.figshare.1126648.

**Figure 4:**
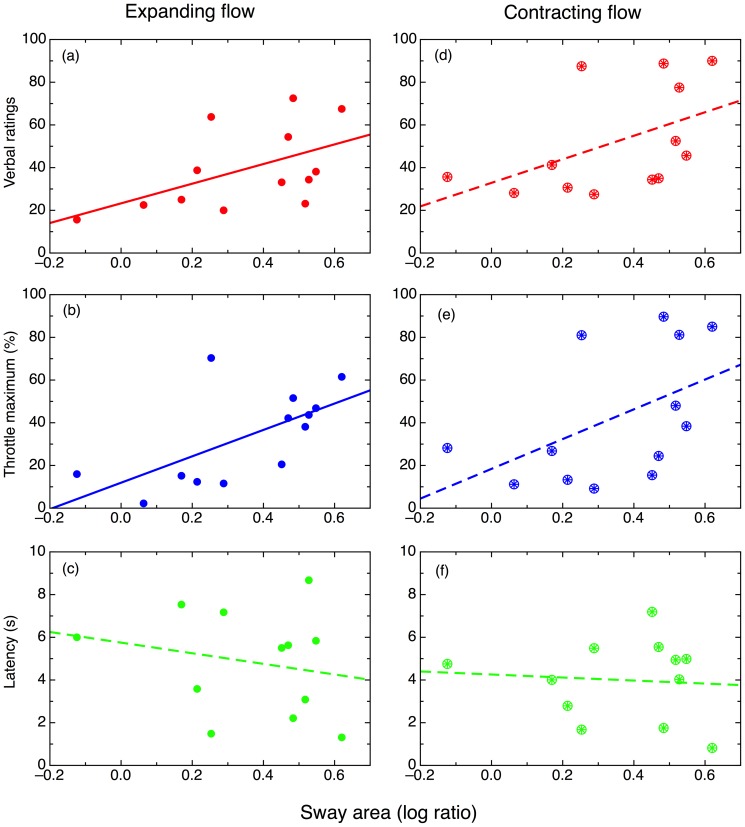
Correlations between vection measures and sway area ratios (log transformed). (a–c) Expanding vection; (d–f) Contracting vection. (a, d) Verbal ratings. (b, e) Throttle maximum values. (c,f) Latency.

Next, we computed the 95% confidence ellipse for sway area, as shown in Figure 3. This calculation was performed using principal component analysis (PCA) to fit the ellipse's semi-axes [48], using Matlab code written by Marcos Duarte, available with permission in our Figshare data repository (http://dx.doi.org/10.6084/m9.figshare.1126648); a Python version of this code is also freely available at http://demotu.github.io/posts/prediction-ellipse-ellipsoid.html. We then investigated whether the ratio of sway area with eyes open and sway area with eyes closed could predict the verbally and manually rated strength of seated vection. This relationship was significant for expanding flow (see Figure 4), for the verbal and throttle maximum measures, but not for contracting flow; latency was not significant for either expanding or contracting flow. (For statistics, see Table 2). This result provides some support for our earlier finding that the Romberg Ratio could predict standing vection, at least for smoothly moving stimuli, in another adult sample [22].

**Table 2:** Correlations between VEPRs and vection.

Measure	Expanding	Contracting
Verbal (%)	.62 (.02)	−.45 (.12)
Throttle max (%)	.78 (.002)	−.41 (.16)
Latency (s)	−.37 (.23)	.61(.04)

Pearson correlations (*r*) and p-values (in brackets) for the relationships between visually-evoked postural responses to expanding or contracting optic flow patterns and subsequently experienced seated vection.

### Visually-evoked postural responses and seated vection

The postural responses of individuals to expanding and contracting optic flow have been shown to be related to, but not directly predictive of, the vection experience during stimulus exposure [12], [18], [19]. Here we asked a different question: could the magnitude of postural response to these kinds of stimuli predict the magnitude of vection an individual would experience in a separate, seated session?

For each expanding and contracting radial flow session, we computed the average anterior-posterior (AP) position during motion stimulus exposure, and compared it to the baseline period immediately before each motion period (to control for long-term postural drift). Mean backward sway during expanding motion was not significantly different to 0 (1.16 mm; SD = 2.7 mm), probably reflecting the fact that, on average, participants tended to correct their initial backward sway, but these corrections were quite variable. Forward sway during contracting motion was substantially larger (8.94 mm; SD = 4.6 mm), and this difference was significant, *t*(12)  =  5.81, p<.001. This is consistent with previous research showing much larger magnitudes for contracting flow, probably due to foot physiology [12]; simply, it is possible to sway much further forward than backward before falling over.

**Figure 5:**
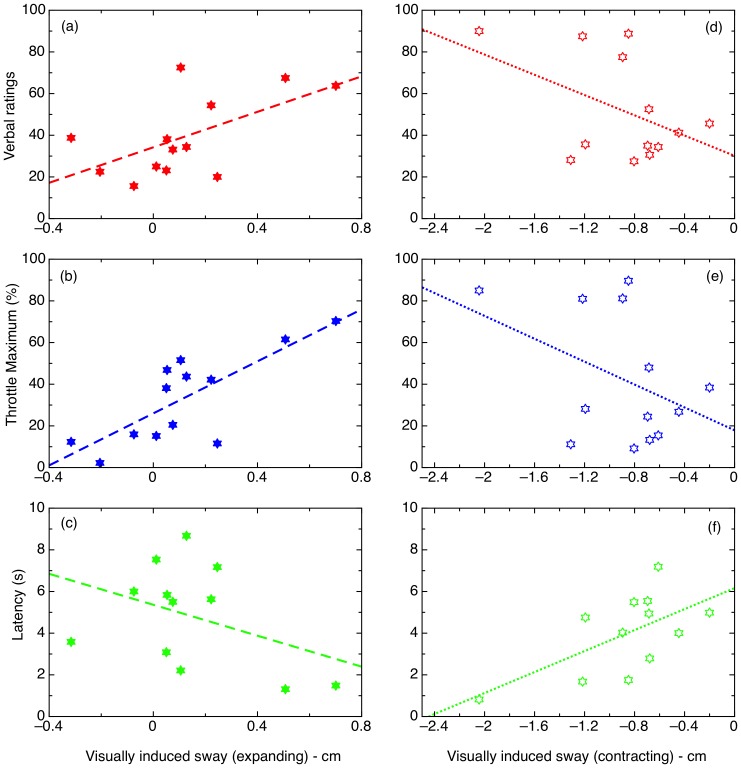
Correlations between vection measures and visually-evoked postural responses. The VEPR was measured as the mean position difference (forward or backward) between a period of optic flow (expanding or contracting) and the preceding period. Top: verbal ratings. Middle: Throttle maximum values. Bottom: Latency. Vection for expanding stimuli is plotted on the right, and for contracting on the left.

We were chiefly interested in whether these measures could predict subsequent seated vection, and indeed they showed a significant relationship with expanding vection for verbal and throttle maximum measures, and for latency during contracting vection (see Figure 5). Contracting sway means did generally not prove to be very robust predictors of vection during contracting sway. These relationships also point to a more complicated, perhaps non-linear relationship between sway magnitudes and vection. Thus it seems reasonable to explore non-linear measures, both of quiet stance and of sway during optic flow exposure, in further detail.

### Recurrence analysis of CoP during quiet stance and seated vection

Given that many other researchers have begun to use nonlinear dynamic approaches to postural sway in such diverse fields as ageing, sport and athletics, diabetes and Parkinson's Disease [3], [6], [31], [32], [47], [49], we wondered if a nonlinear analysis of the recurrent patterns in postural control might give us more insight into the relationship between postural control and vection. We chose recurrence quantification analysis (RQA) because of its relative simplicity and widespread use throughout the literature.

The parameters used in our analysis were similar to those used in [45], since our methods (e.g. time period, eyes open and closed conditions) were very similar; we used an embedding dimension of 8, a delay (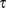) of 15, a radius of 30 and a line minimum of 4. Prior to analysis, the raw data was smoothed by averaging across 10 data points, removing high-frequency noise and rendering the time series more tractable for analysis.

**Figure 6:**
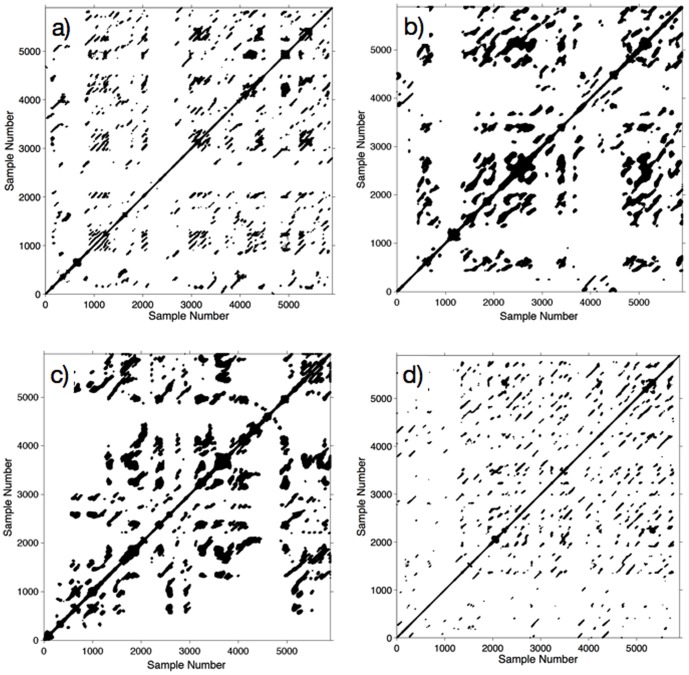
Representative recurrence plots for eyes-open and eyes-closed conditions for two individuals. a) Eyes-open for an individual who experienced strong vection. b) Eyes-closed for the same individual c) Eyes-open for an individual who experienced weak vection d) Eyes-closed for the same individual.

**Figure 7:**
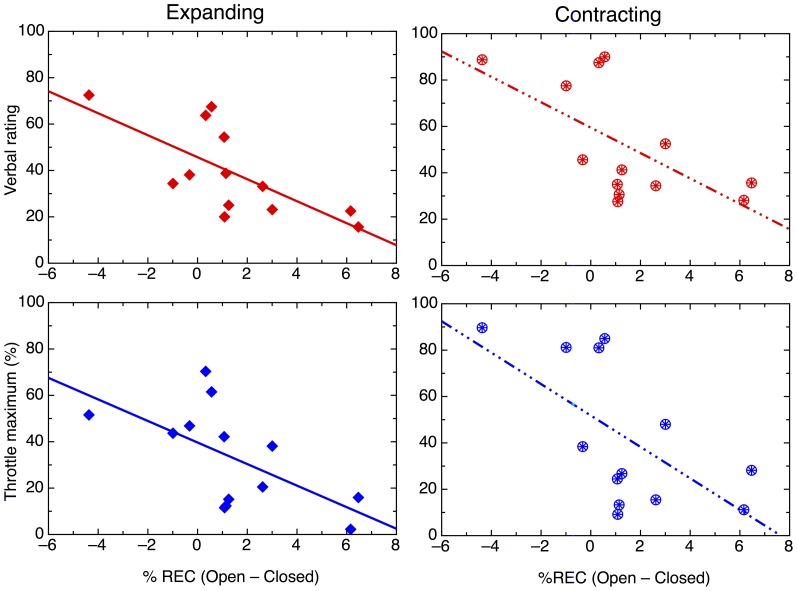
Correlations between vection measures and the *difference* between % recurrence for the quiet-stance eyes open and eyes closed, as measured by RQA. The percentage of recurrence was measured using the recurrence quantification Maltab toolbox, downloaded from http:/nuweb.neu.edu/cjhasson. This means that individuals who experienced stronger vection showed a greater percentage of recurrences with eyes closed than with eyes open, while the reverse was true for those who experienced weaker vection.

Examples of recurrence plots for the anterior-posterior sway time series from two individuals are shown in Figure 6. Eyes-open plots are shown on the left; the individual in the upper part of the figure (Figure 6a and b) experienced strong vection, while the individual in the lower part (Figure 6c and d) experienced weak vection. Essentially, the individual who experienced strong vection displayed a higher percentage of recurrence in the postural sway time series with the eyes closed compared to open; the reverse was true for the weak-vection individual. This pattern persisted across the entire group, for both expanding and contracting vection, as shown in Figure 7. Interestingly, the eyes-open data alone predicted seated vection quite robustly (correlations are reported in full in Table 3. This suggests that individuals who experience stronger vection show fewer recurrences in their patterns of postural sway when standing quietly with their eyes open (i.e. experiencing visual feedback on their postural stability). The implications of this will be examined further in the Discussion.

**Table 3:** Correlations between percent recurrence (RQA) and vection.

Measure	Eyes open - eyes closed	Eyes open	Eyes closed
Verbal expanding (%)	−0.70 (.008)	−0.74 (.004)	0.20 (.5)
Throttle max expanding (%)	−0.61 (.026)	−.72 (.005)	.08 (.79)
Verbal contracting (%)	−0.63 (.02)	−0.62 (.02)	0.25 (.41)
Throttle max contracting (%)	−0.62 (.02)	−.59 (.04)	.28 (.35)

Pearson correlations (*r*) and p-values (in brackets) for the relationships between the chosen RQA measure (percentage of recurrences) for eyes-open compared to eyes-closed, eyes-open only and eyes-closed only data, with verbal and throttle measures for expanding or contracting seated vection ratings.

Other measures from the RQA analysis, such as percent of determinism and laminarity, were also significantly correlated with the vection measures. These relationships are outlined in full in the Supplementary Data available on Figshare (http://dx.doi.org/10.6084/m9.figshare.1126648).

## Discussion

We set out to explore the role of visual control of posture in determining individual variations in the experience of vection. Overall, we found the three out of four of our measures of postural control while standing (sway area ratio, VEPRs and recurrence rate) predicted individual variations in the experience of seated vection. Importantly, all of these measures were concerned with the influence of *vision* on postural control - none of the eyes-closed measures alone predicted vection. However, we do not suggest that vision alone is the arbiter of the vection experience - rather, it is the complex interaction between the visual system and other systems governing postural control, such as vestibular and proprioceptive systems, that is at work here, and our proxy for investigating these interactions was variation in the CoP during both quiet stance and visual optic flow.

Brain imaging studies have implicated a number of visual cortical areas in self-motion processing, including the medial temporal area (MT/V5) [50], the medial superior temporal area (MST) [51] and its dorsal subdivision (MSTd) [52], the dorsomedial area (V6) [53], [54], the cingulate sulcus visual area (CSv) [55], and the ventral intraparietal area (VIP) [56]. However, vestibular and multisensory areas of the cortex have also been implicated, including the intraparietal sulcus motion area (IPSmot) [56], the parieto-insular vestibular cortex (PIVC) [57] and putative area 2v (p2v) [58], as well as the precuneus motion area (PcM) [59]. Although only a handful of these studies have explicitly measured vection, it seems likely that a network of brain areas are involved in self-motion perception, and this network almost certainly involves feedback, which points to complex non-linear interactions between these brain areas.

Although we found reasonable predictions for both independent linear measures for seated vection during expanding optic flow, we were puzzled by the lack of any reliable prediction for contracting flow. As reported above, verbal vection measures were significantly higher for contracting than expanding flow, although not for throttle maximum or latency. Interestingly, in our previous study, we also found that linear measures of postural sway (Romberg ratios) were only able to predict the less-compelling vection induced by smooth optic flow (compared to jittering flow). Could it be that sway measures are only informative in the case of weaker vection? Or is it possible that there are more complex interactions involved in the relationship between postural control and the experience of vection which are not well captured by linear measures?

Interestingly, the non-linear measures proved more predictive of subsequently experienced seated vection than the linear measures, particularly with regard to quiet standing; this is in line with previous research suggesting fractal measures of quiet stance are more reliable [60]. Another interesting aspect of this data is that the eyes-open measures alone predicted vection strongly (see 3); this was not the case for sway area or path length, perhaps because these linear measures incorporate absolute sway magnitude, which can vary with individual attributes such as height, weight and foot width, which are unlikely to be related to the tendency to experience vection. Non-linear measures are more related to the underlying structure of the data than to global variations [30], [36], [45], [60]. This also suggests that the assumptions underlying linear measures of postural control may be flawed, perhaps undermining their robustness as predictors.

Much of the literature on recurrence analyses of postural sway, in both adults and children, has been concerned with the effects of injury, disease or ageing [3], [31], [61], [62]. Older adults in particular show a pattern of fewer recurrences during quiet stance [43], and this has also been shown to predict falls in older adults [3]. However, our sample was uniformly young and healthy (mean age  = 20.9, SD = .76, range  = 20–22, no reported injures, disabilities or vestibular issues). To our knowledge, this is the first report of recurrence measures reliably predicting a behavioural measure in such a sample. It is worth noting that, for a broader age range, age should be controlled for in this relationship.

We found stronger vection overall for contracting than for expanding optic flow, at least for the verbal measures, and also greater sway magnitude (both for mean anterior-posterior sway and for path length during optic flow in comparison to fixation). However, the linear measures we used (Romberg ratio, sway area ratio and VEPRs) all failed to predict individual variations in the magnitude of vection in response to contracting stimuli. It seems unlikely that this was due to ceiling effects in the data, as the maximum average verbal vection report in this condition was 93%. The nonlinear measures alone provided reliable predictions of contracting vection strength; their predictions for expanding vection were also considerably stronger than most of the linear measures.

It is possible that the asymmetry between predictions for expanding and contracting vection could arise because multisensory processing during expanding and contracting optic flow differs; the postural adjustments for the two different types of real-world situations (for instance, falling forwards compared to falling backwards) may rest on different sensory weightings and different levels of feedback between sensory systems.

Expanding flow occurs during forward motion, which is key for moving around the environment and thus requires very fine control. Contracting flow occurs during backward motion or falling, which are rare and may require fast, reflex-like responses involving more coarse processing. If falling backwards is dealt with by systems optimised for reaction speed and not precision, they may depend more on vestibular input and less on visual input. Vestibular information is easier to process than visual information, so much so that it instigates the Moro reflex to lack of support in newborns [63]. Fast processing may thus attend more to the vestibular system, explaining the lack of correlation between vection and sway under contracting optic flow.

Overall, it is clear that non-linear dynamical analysis (RQA) of postural sway during quiet stance can provide useful predictions about an individual's likelihood of experiencing illusions of self-motion. This could prove a useful tool for evaluating individuals before participation in virtual reality experiments, flight simulation training, and so on. In future, it would be fascinating to explore the possibility of classifying individual EEG data during vection compared to no-vection states, to explore whether RQA could be equally useful in examining neural state changes related to vection.

## Materials and Methods

### Participants

Participants were 13 healthy third-year undergraduate students who volunteered as part of a course assignment. Mean age was 20.9, mean height 171.7 cm, and mean mass 75 kg; three participants were male. (Full demographic data is available at http://dx.doi.org/10.6084/m9.figshare.1126648).

We note that, though gender was not evenly balanced, it has been reported that there are no gender differences in vection [64]; indeed, we confirmed this finding across a combined sample of this dataset and a previously published experiment [22] (N = 33, n(male)  = 7); neither verbal nor throttle measures, nor any of the sway measures, were significantly different between genders (all p-values>.1), though expected measures such as height (p<.001) and foot length (p<.001) did show highly significant gender differences, indicating that there was sufficient power to detect any differences.

### Ethics Statement

The experiments were approved by the Human Ethics Committee of the University of Wollongong (approval number HE10/120). All participants gave informed written consent and were free to withdraw from the study at any time if they experienced discomfort or motion sickness. The study conformed to the guidelines set out in the Declaration of Helsinki.

### Apparatus

#### CoP acquisition

Postural sway data was measured with a Bertec Balance Plate, using Bertec Acquire 4 software (Version 4.0.11.312) connected to a Dell Optiplex GX620 computer, running Windows XP. The data were sampled at 1000 Hz and recorded in a Matlab file; for analysis, the data were smoothed with an order 5 Butterworth filter to remove low-frequency artefacts. During the quiet stance and standing vection conditions, the plate was positioned 65 cm from the screen.

#### Seated vection ratings

During the seated vection conditions, subjects viewed stimuli through black-lined viewing tube fronted by a rectangular black cardboard frame, to give a field of view of 44 degrees horizontally and 26 degrees vertically, and were seated in front of the tube. The viewer was positioned 153 cm from the screen, with his or her face aligned with the back of the viewing tube. During these conditions, as well as giving verbal ratings at the end of each vection trial, participants rated the strength of vection using a USB throttle device (CH Pro USB throttle), which sampled its position at a rate of 100 Hz.

### Stimuli

Optic flow stimuli were generated and displayed separately using Matlab version R2009b, running on a Mac Pro computer (Mac Pro 3.1, Quad-Core Intel Xeon 2.8 GHz) and the Psychophysics Toolbox [65], [66], and displayed using a Mitsubishi Electric colour data projector (Model XD400U) back-projected onto large (1.48 m wide by 1.20 m high) screen mounted on the lab wall. Stimuli were random clouds consisting of 1000 blue circular dots, moving in a radially expanding or contracting fashion (see Movies S1 and S2), within a virtual “world”, 30 by 30 by 80 m in virtual units. The dot cloud moved at a simulated self-motion speed of 6 m/s, and either expanded towards or contracted away from the observer in separate sessions (see Procedure).

### Procedure

Before the main experiment, participants filled in some basic demographic information and completed the first part of Kennedy's Simulator Sickness Questionnaire [67]. After this, a few basic physiological measures (height, weight, foot length, foot width) were taken. We then obtained CoP recordings without and with vection, followed by vection ratings from a seated position.

During the initial quiet stance conditions, the room was brightly lit to ensure ample visual cues for postural control, but the screen remained blank.

During the optic flow conditions, the room was darkened and external sources of light were minimised by turning off the external monitor, all other lighting sources, and, during seated vection conditions, covering the participant's head with a black cloth draped around the viewing tube.

At the end of the experiment, participants filled out the last section of Kennedy's Simulator Sickness Questionnaire, to give a post-experiment measure of motion sickness.

#### CoP acquisition

Participants were asked to stand on the balance plate with their ankles aligned with the plate markings, with their feet together. Foot position on the plate was marked with erasable marker to ensure position was maintained if participants needed to step off the plate between sessions. They were instructed to stand with hands folded in front of them and gaze straight ahead. Then participants were instructed to stand as still as possible with eyes either closed or open (this was counterbalanced to eliminate order effects) while their CoP movement was recorded for 60 seconds.

After a break, participants returned to the balance plate, standing in the same position as in the quiet stance trials, delineated by the markings on the plate. The observer was now 65 cm from the screen, giving a field of view of 66 by 62 degrees of visual angle, and the stimuli were adjusted accordingly. There were two sessions, again with expanding and contracting stimuli blocked. Each session consisted of a 30 s blank period, followed by 30 of the optic flow stimulus, 30 seconds of a blank screen, and 30 seconds of a simple fixation screen with instructions to continue standing steady; this sequence was repeated three times, and postural sway was again recorded for the entire session as outlined above.

#### Seated vection ratings

Following this, the seated vection conditions were run. Participants were seated on a raised architect's chair, with feet resting on a metal ring at the base of the chair, and head just inside the viewing tube as described above. After being given a basic description of vection, participants were asked to move the throttle forwards during the vection display, if and when they felt that they were moving, to rate the extent to which they felt they were moving (and specifically not the speed of their self-motion), and to move it back if they felt they were moving less or had stopped moving; the device had tactile marking points (small raised bumps at 0, 50 and 100% positions), to assist participants in rating vection strength. The computer was programmed to require the throttle to be reset to 0 before the next trial could proceed. After each trial, participants were also asked to also give a verbal rating of their vection experience, from 0 (no self-motion) to 100 (complete self-motion); this was followed by a blank period of 5 seconds to help reduce any residual effects of adaptation. Each participant completed 8 trials of each stimulus type (expanding or contracting), and these were blocked and counterbalanced between participants to avoid order effects.

## Supporting Information

Movie S1 **A demo movie of the expanding vection-inducing optic flow stimulus.** Note that this does not precisely represent the size and optic flow speed of the stimulus due to refresh rate and screen size differences. The original stimulus was presented on a large wall-sized screen.
(MOV)

Movie S2 **A demo movie of the contracting vection-inducing optic flow stimulus.** Note that this does not precisely represent the size and optic flow speed of the stimulus due to refresh rate and screen size differences. The original stimulus was presented on a large wall-sized screen.
(MOV)
